# Women Runners in China: Constraints Negotiation Process of Serious Leisure

**DOI:** 10.3390/ijerph19010214

**Published:** 2021-12-25

**Authors:** Wenting Zhou, Yajun Qiu, Haibo Tian, Jiao Xu

**Affiliations:** 1 Department of Physical Education, College of Education, Zhejiang University, Zijingang Campus, No. 866, Yu Hang Tang Road, Hangzhou 310058, China; zipig12321@163.com (W.Z.); qiuyajun@zju.edu.cn (Y.Q.); 2 School of Teacher Education, Shaoxing University, 508 Huan Cheng Xi Road, Yuecheng District, Shaoxing 312000, China; thbzhy@163.com; 3 Zhejiang University City College, No. 48 Huzhou Street, Hangzhou 310015, China

**Keywords:** women runners, constraints, negotiation, serious leisure, intrinsic motivation

## Abstract

The constraint negotiation process is a prominent part of serious leisure, and leisure-oriented women runners in China may behave differently in this process. An adjusted model was proposed to examine the constraint negotiation process of serious leisure for women runners. An online questionnaire was conducted that contained 239 valid samples measuring the participation, intrinsic motivation, constraints and negotiation of women runners. The structure of variables was confirmed based on the good results of reliability and validity test. Then the structural equation modeling results showed that constraints had a negative impact and negotiation had a positive impact on participation. Negotiation acts on constraints to reduce their negative perception. Furthermore, intrinsic motivation has a significant positive effect on negotiation. There are high intrinsic motivation and fewer constraints reporting for women runners under high negotiation in serious leisure. The results provide additional explanation for the serious leisure participation of women runners. Future research should integrate women’s life experiences to better understand the behavior revealed in this study.

## 1. Introduction

Research on women and leisure emerged approximately 40 years ago [1,2]. While more researchers have searched for a universal explanation, Henderson [3] pointed out that one size doesn’t fit all and the development of society is markedly influenced in women’s leisure. China has made great progress in terms of gender equality in the past, but because gender profoundly affects all areas of people’s lives, the gap in leisure sports still exists in the lives of men and women [4].

In many leisure sports, running, which is simple and easy to participate, has recently become popular in China. According to government data, in 2016, 2017 and 2018, 328, 1102, and 1508 marathon events were held nationwide, respectively, while the number of participants more than double, from 2.8 million to 5.83 million. However, among numerous marathon participants, women runners accounted for less than 30%, including 27.2% in 2017 and 26.9% in 2018 (China Athletics Association). Although women have started to more strongly pursue deeper experience in leisure and regarded long-term involvement in running as serious leisure, they are more susceptible to constraints than men are [5].

Serious leisure is defined as ‘the systematic pursuit of an amateur, hobbyist, or volunteer core activity that people find so substantial, interesting, and fulfilling that, in a typical case, they launch themselves on a (leisure) career centered on acquiring and expressing a combination of its special skills, knowledge, and experience [6]. Compared with instant and casual leisure such as chatting and shopping, serious leisure often requires participants to spend a lot of time and energy to pursue a deeper experience. For example, a serious leisure runner will develop a detailed plan to improve his running ability, get support and encouragement from the leisure group, and then gain a series of positive experience and benefits from the enduring involvement [7]. Six qualities of serious leisure participants have been generalized [8,9]. They would make significant personal efforts to learn skills and knowledge and need to persevere involved to experience durable benefits. At the same time, they share a unique ethos with participants in the social world and strongly identify with activities and themselves through the leisure career.

Stebbins [6,9] mentioned the need to persevere as “conquering adversity” or “sticking with it through thick and thin”. Serious leisure would give the individual a great variety of experiences, in which the perception of constraints is inevitable [10,11]. There will be unfavorable situations, such as injury and disappointing. For continuous participation, it is essential for them to adopt effective negotiation ways to deal with unfavorable conditions [12,13]. Stebbins [9] believed that the individual’s sense of self-satisfaction and self-realization will be more perceived after overcoming difficulties. Increasing research has focused on female participants in serious leisure [14,15]. It is obvious that women will obtain various positive benefits from serious leisure activities and promote their continued participation [15,16]. Moreover, empirical evidence suggests that women may experience different serious leisure careers, and gender differences in social roles or others may hinder this process [14,17,18].

Leisure constraints are defined as “anything that inhibits people’s ability to participate in leisure activities, to spend more time doing so, or to take advantage of leisure services, or to achieve a desired level of satisfaction” [19,20]. Many studies used the classification according to Crawford and Godbey [21], dividing leisure constraints into intrapersonal (e.g., injuries, lack of confidence), interpersonal (e.g., lack of partner) and structural (e.g., lack of money, poor weather) constraints. There may be a hierarchical influence on leisure participation among the three types of constraints, which intrapersonal faced first, then interpersonal, and structural last [22]. With deeper awareness of constraints, researchers have indicated that constraints do not entirely hinder leisure participation, and their negative impact is reduced after successful negotiation [23,24].

Leisure negotiation refers to the strategies adopted by individuals to avoid or reduce the impact of constraints on leisure participation and leisure enjoyment [25]. Primary categories of negotiation strategies were behavioral (e.g., gaining professional knowledge, changing leisure activities) and cognitive (e.g., reducing cognitive dissonance, improving persistent commitment) initially [26]. Then, the different categories of negotiation strategies used, such as time management, skill acquisition, interpersonal coordination and financial resources [27], or six negotiation strategies based on qualitative and quantitative research in leisure sports activities in China were named consciousness-raising, self-management, helping relationships, time management, item adjustment, and supportive environment [28]. The positive effect of negotiation explains the situation in which people continued participation despite having constraints. Alexandris et al. [29] showed that swimming amateurs with high levels of participation scored the highest in negotiation.

Jackson et al. [23] also stated that the negotiation process depends on the relative strength of motivation to participate. Iso-Ahola [30] indicated that intrinsic motivation is the basis of individual leisure behavior. Self-determination theory is generated by an individual’s interest or pleasure in the activity itself [31], which is usually accompanied by the experience of positive emotions, flexibility and choice and could promote the continuity of leisure behavior [32]. Frederick-Recascino and Schuster-Smith [33] showed that the level of intrinsic motivation was positively associated with exercise behavior. Santos et al. [34] found that intrinsic motivation matters in sustaining leisure-time physical activity participation for women.

The process of constraint negotiation has triggered a series of studies to focus on [35,36,37]. Hubbard and Mannell [27] proposed four models testing the relationship between constraints, negotiation, motivation and participation. The results supporting the constraint-effect-mitigation model gained the most verification by other researchers [38,39]. It showed that constraints will promote the use of negotiation strategies and have a negative effect on participation, while participants with higher motivation are more likely to adopt negotiation strategies to maintain participation. However, some researchers have found different results on this model; for example, the relationship between constraints and negotiation was not significant [40,41], and negotiation had less effect on participation [42]. Meanwhile, other variables were further included in the constraint negotiation process, such as negotiation efficacy [42,43], identity [44], and involvement [45].

With the different results, further testing is needed based on the serious leisure participation of women runners. Serious participants had a higher ability to take kinds of negotiation strategies to adapt or mitigate constraints [46]. Women runners of serious leisure always pursue long-distance running ability and regard achieving a marathon as a milestone. The quality of perseverance is obviously represented in women runners who make efforts in marathon running, including success in negotiating constraints [47]. Qualitative studies have shown that the significance of serious leisure for women far exceeds the leisure activity itself, especially since successful negotiation constraints could endue women from accepting deeper experiences [48]. Ridinger et al. [49] also found that women runners had stronger negotiation efficacy than men. Hence, more emphasis should be placed on the effect of negotiation on constraints in serious leisure.

To date, increasing attention has been paid to people’s sports participation in serious leisure, especially female participants [16,17,48,50], but the constraint negotiation model has rarely been examined for women runners of serious leisure. Therefore, this study aimed to examine the constraint negotiation process of Chinese women runners in serious leisure based on an adjusted model. We expect that these results could provide guidance to help women better understand their leisure experiences and leisure service providers to pursue appropriate service improvements.

Previous studies have suggested that serious leisure participants put great effort into facing constraints, which would strengthen the effect of negotiation on constraints. Additionally, intrinsic motivation had a greater influence on continued leisure sports participation. Thus, we believe that the constraint negotiation process for women runners of serious leisure would be more in line with the perceived constraint reduction model proposed by Hubbard and Mannell [27]. The following hypotheses are tested in our model:

**Hypothesis** **1** **(H1):** *Constraints to women runners have a negative effect on participation in serious leisure.*

**Hypothesis** **2** **(H2):** *Negotiation to women runners has a positive effect on participation in serious leisure.*

**Hypothesis** **3** **(H3):** *Intrinsic motivation for runners has a positive effect on participation in serious leisure.*

**Hypothesis** **4** **(H4):** *Negotiation to women runners has a negative effect on constraints in serious leisure.*

**Hypothesis** **5** **(H5):** *Intrinsic motivation for women runners has a positive effect on negotiation in serious leisure.*

## 2. Method

### 2.1. Participants

Purposive sampling was used to select women runners who reported having participated in at least one marathon or half marathon. A total of 254 online questionnaires were recovered through a web-based questionnaire platform from November to December 2019, of which 239 were valid. All respondents between 20–55 years old, 40.6% in the age bracket of 20–29 (*n* = 97), 44.4% in the age bracket of 30–44 (*n* = 106), and 15.1% in the age bracket of 45–55 (*n* = 36). The marital status of nearly half of the respondents was married (*n* = 117) and unmarried (*n* = 108), others were divorced or living alone (*n* = 14). The majority had at least a college or university degree (*n* = 224). More than half of runners had joined a running group (*n* = 159) and had an annual income above $7500 (*n* = 169), which is above the national average income level (i.e., US$3969).

### 2.2. Measurements

Participation was measured using the product of three items. The first item is the number of running years that specifies “1” for 6 months ≤ time < 1 year, “2” for 1 year ≤ time < 3 years, “3” for 3 years ≤ time < 5 years, and “4” for time ≥ 5 years. The second item is the running frequency per week, which specifies “1” for 1–2 times, “2” for 3–4 times, and “3” for 5 times or more. The third item is the distance per run that specifies “1” for distance < 5 km, “2” for 5 km ≤ distance < 10 km, “3” for 10 km ≤ distance < 20 km, and “4” for distance ≥ 50 km. Participation = number of running years * running frequency per week * distance per running.

Intrinsic motivation was measured by adopting the Sport Motivation Scale-6 (SMS-6) [51], which is based on self-determination theory and has undergone a series of developments [52,53]. This study chose to focus on intrinsic motivation (4 items) and modified the context of China and running. For example, “For the pleasure of discovering new performance strategies” was adapted to “For the pleasure of acquiring new running knowledge and skills”. A five-point Likert scale ranging from strongly agree (5) to strongly disagree (1) was used.

Constraints were measured using a homemade questionnaire for three types [21]: intrapersonal (5 items), interpersonal (3 items), and structural (4 items). The 12 items were identified by referring to previous studies on leisure sports [40,54,55] and by developing new items for running. For example, “Physical fatigue from running” was added because this often occurs during a runner’s participation; for example, they describe “hitting the wall”, resulting in negative effects. A five-point Likert scale ranging from strongly agree (5) to strongly disagree (1) was used.

Negotiation was measured based on previous research, combining the two studies of Qiu [28] and Zhou et al. [13] and creating five types of negotiation strategies: consciousness-raising strategies (3 items), self-management strategies (3 items), helping relationships strategies (4 items), time adjustment strategies (3 items), and supportive environment strategies (3 items). Furthermore, some items were modified for running. For example, “Meet people who like sports” was adapted to “Communicate with experienced runners”. A five-point Likert scale ranging from strongly agree (5) to strongly disagree (1) was used. The reliability coefficient of the time adjustment strategy was low (α = 0.510), so it was deleted and no further analysis was performed.

### 2.3. Data Analysis

This study used Statistical Product and Service Solutions (SPSS 25.0) for the primary data analysis, including descriptive statistics, reliability and correlation of variables. Then, AMOS 24.0 was used to test the model of the constraint negotiation process. For intrinsic motivation, constraints and negotiation, confirmatory factor analysis (CFA) was used to confirm the factor structure. Based on the factor loadings provided by the standardized parameter estimation (*β*), the average variance extracted (AVE), composite reliability (CR), and discriminant validity were calculated. AVE greater than 0.50 and CR greater than 0.70 were used as criteria of a good fit. Considering the actual aspect of the data, AVE between 0.36–0.50 was also acceptable [56].

When the structure of variables was confirmed, the structural model proposed in this study was tested to estimate the model fit. Bollen [57] suggested using multiple fit indices to evaluate model fit, including nonsignificant chi-square (χ^2^), root mean square of approximation (RMSEA) less than 0.05, goodness-of-fit index (GFI), adjusted goodness-of-fit index (AGFI), normed fit index (NFI), and comparative fit index (CFI) greater than 0.90. However, a nonsignificant chi-square statistic was difficult to achieve because it is sensitive to sample size and magnified type I errors [58]. Therefore, the ratio of chi-square to degrees of freedom (χ^2^/df) was used, and ranging from 1 to 3 was acceptable fit [59].

## 3. Results

### 3.1. Descriptive Results

The mean score of respondents’ participation was 11.435, with a minimum of 1 and a maximum of 45. Respondents had high intrinsic motivation (M = 4.077) to participate in running. They showed moderate constraints (M = 3.120), with all subentry mean values near 3. The highest influence was intrapersonal constraints (M = 3.237), followed by structural constraints (M = 3.165) and interpersonal constraints (M = 2.928). For specific items, “Physical injury (M = 3.757)”, “Unfavorable weather (M = 3.439)”, and “Physical fatigue from running (M = 3.385)” obtained the highest scores. Furthermore, respondents had a high level of negotiation strategies (M = 3.791), with subentry mean scores ranging from 3.548 to 3.975. They used consciousness-raising strategies (M = 3.915) the most and supportive environment strategies (M = 3.667) the least (see Table 1). For specific items, “Strengthen willpower (M = 3.975)”, “Scientific training to improve running skills (M = 3.929)”, and “Set goals (M = 3.908)” obtained the highest scores.

### 3.2. Reliability and Validity

The findings in Table 1 revealed that the internal consistency of each construct measured by Cronbach’s alpha was between 0.792–0.935, which is close to or greater than 0.80, indicating good reliability of the factor structure [60]. Then, AVE (from 0.470 to 0.724) and CR (from 0.787 to 0.913) showed acceptable levels for all factors, suggesting good convergent validity. Although the AVE values of intrapersonal constraints and structural constraints were lower than 0.50, they were within the acceptable range of 0.36–0.50 [56]. In Table 2, discriminant validity affirmed that the square roots of the AVE values of the variables were greater than the correlations between the other two variables [61].

### 3.3. Structural Model

**Figure 1:**
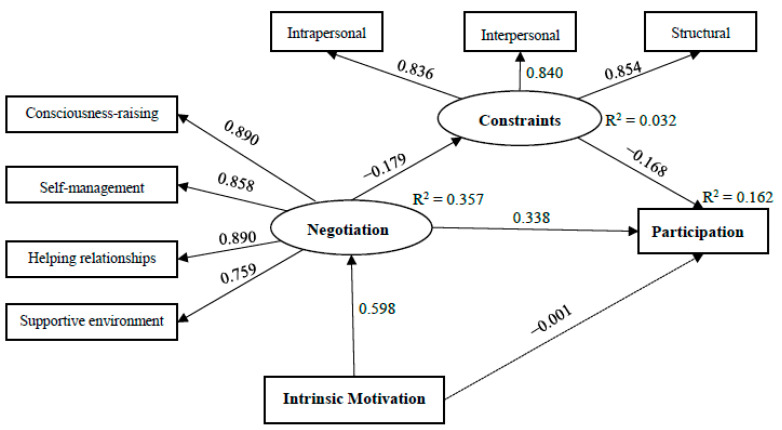
Structural model of the constraint negotiation process with standardized parameter estimates. Note: Broken line means the relationship of two variables is not significant at the 0.05 level.

As shown in Figure 1, the model of leisure constraint negotiation proposed in this study tested with structural equation modeling could adequately fit the data (χ^2^/df = 1.589, RMSEA = 0.050, GFI = 0.964, AGFI = 0.932, NFI = 0.969, CFI = 0.988). Participation was negatively affected by constraints (*β* = −0.168, *p* < 0.05) and positively affected by negotiation (*β* = 0.338, *p* < 0.001), as predicted (H1 and H2). These two variables account for 16% of the variation in the level of participation. Constraints (*β* = −0.179, *p* < 0.05) were negatively predicated by negotiation (H4), accounting for 3% of the variance. This finding reflected that respondents’ perception of constraints would be reduced by the use of negotiation strategies. Intrinsic motivation (*β* = 0.598, *p* < 0.001) had a significant positive effect on negotiation (H5), accounting for 36% of the variance. This indicated that respondents driven by intrinsic motivation would more actively negotiate constraints. However, intrinsic motivation (*β* = −0.001, *p* > 0.05) did not have a significant effect on participation (H3) in this model.

## 4. Discussion

The purpose of this study was to assess the constraint negotiation process of women runners in serious leisure. As theory has suggested, serious leisure pursuit has been closely associated with internally committing to activities and investing effort to conquer problems during long-term participation [6,11]. The results revealed that women runners perceived some constraints and had high negotiation. The intrapersonal constraints of physical influence were most influential, which is consistent with previous studies showing that individuals who have long been involved in running will face physical pain and the process of coping pain [62]. Women’s runners had high negotiation scores on raising consciousness and self-management. They would strengthen their willpower and scientific training to insist and avoid injury. In addition, women runners are easily affected by the weather of structural constraints. This is an uncontrollable factor, sometimes requiring them to change the running location. Through long-term participation, women runners could accumulate rich experience in negotiating constraints and would not feel a strong sense of hindrance when encountering difficulties next time.

Building from the existing literature, the perceived constraint reduction model proposed by Hubbard and Mannell [27] was adjusted for this study to illustrate the connection of constraints, negotiation, intrinsic motivation, and participation. The five hypotheses of association were empirically tested by using structural equation modeling. The results provided partial support for the proposed model. It showed first that women runners of serious leisure participation were negatively affected by constraints and positively affected by negotiation. Constraints were conceptually expressed as a negative influential factor, and negotiation could be seen as coping resources directly forcing participation [63]. For example, when an increasing number of women have their own jobs, they undertake the double burden of work and family affairs. The structural constraint of lack of time often makes it difficult for many women runners to maintain running, but serious female runners always work harder to increase willpower, and to obtain support from their families and groups to find time to run and improve the quality of leisure.

The constraint-negotiation relationship in this study showed that negotiation had a significant negative impact on constraints. This finding supported the hypothesis of the perceived constraint reduction model that ‘people with sufficient negotiation resources will perceive themselves to be less constrained’ [27]. The quality of need to persevere demonstrated the remarkable negotiation ability of serious runners to actively reduce the effects of leisure constraints [5]. Judging from the proportion of females with an increasing participation base in China, an increasing number of women runners appeared. Although women experience more constraints related to physiological differences, family responsibilities and so on, they could use negotiation strategies, such as joining a group, or learn skill knowledge to overcome difficulties [36,54,64,65]. The perception of constraints would be moderate by the experience of successful negotiation constraints [13]. Moreover, with the development of society in China, the opportunity and authority of women increased. Spending more time in leisure sports activities in turn stimulates women’s subjectivity towards conscious practices, bringing their freedom of choice and empowerment of the body [66]. It is obvious that significant effort in negotiating constraints during serious leisure time was the manifestation of women’s self-challenge.

The study also sheds light on the function of intrinsic motivation in the constraint negotiation process for serious women runners. As predicted, women runners with higher intrinsic motivation have an upper level of negotiation. Intrinsic motivation emphasizes the individual’s inherent tendency to spontaneously participate in an activity [32], which is related to the powerful sense of identity characterized in serious leisure participants. Continuous identity shows the participants’ strong emotional connection to the activity and in generating momentum towards running in the face of disruption [67]. Although many studies have shown that women’s motivation in leisure sports is for external body image [68], our results suggest that women in serious leisure pay more attention to their own feelings and regard running as an important part of their lives. The intrinsic driving force will further encourage participants who wonder deeper leisure experiences to be involved in and motivate them to overcome difficulties. Moreover, the effect of intrinsic motivation on negotiation displayed the improvement of women’s self-consciousness and their self-demand to drive the behavior to overcome constraints. Such as, due to the inherent love of running, serious women runners will actively learn scientific training methods to adjust themselves to keep participating when experiencing physical fatigue during running.

In addition, intrinsic motivation and participation had significant correlation estimates, but there was no linking between the two reported in the model. This may be caused by the over-prominence of the direct impact of negotiation on participation. Various leisure constraints, especially gender-related constraints, are inevitable in the process from the motivation to the occurrence of behavior and to serious involvement of women runners, so successful negotiation plays an important role in this process. For example, most women are worried about the safety when going out for running, but outdoor running is more comfortable and attractive, so it is a very important negotiation strategy for them to find one or more companions to accompany. The result is more proof of motivation as a potential trigger of the constraint negotiation process [42,43].

There are some theoretical and practical implications in this study. In terms of the theory, our findings expanded to a part of gender study in serious leisure and leisure constraints, highlighting women’s initiative to negotiate constraints in serious leisure. This study also supported the role of intrinsic motivation in the constraint negotiation process, extending previous research. Furthermore, the verification of the model could promote the understanding of women’s serious leisure and leisure constraints. For practical implications, on the basis of understanding the constraints women runners may encounter, leisure organizations should create more space to not only provide the opportunities for women to learn running knowledge about the injury appeared or scientific skill practice but also organize some easy activities to show the positive benefits of running, such as running daily attendance/clock in, term running relay, and then naturally increase women’s internal drive.

The study has the following limitations. It is a cross-sectional study that measured the leisure constraint negotiation model at a random time point. However, from the quality of career in serious leisure theory [9], women’s experiences with leisure are complex and fall along a continuum [69]. At the same time, the perception of leisure constraints that the individual reported at any moment may not include the constraint factors they have negotiated. Hence, a longitudinal research design is recommended to observe the constraint negotiation process in the future. Furthermore, the meaning of serious leisure sports activities for women can be extended to provide insight into their whole life [48], including not only the internalization of motivation but also the improvement of subjective well-being and life satisfaction. Future research may deeply discuss the meaning of women’s leisure participation.

## 5. Conclusions

This study explored the constraint negotiation process of serious women runners in the Chinese context. Women runners reported moderate constraints and high negotiation. The five hypotheses examining the relationship among constraints, negotiation, intrinsic motivation, and participation were partially confirmed in an adjusted model. The results supported previous literature on the effect of constraints on participation negatively and negotiation on participation positively. There are fewer constraints reporting for women runners under high negotiation in serious leisure. Additionally, the results revealed the significant effect of intrinsic motivation to negotiate in serious leisure, contributing to the literature. The findings thus provide a more structured understanding of women’s constraint negotiation in serious leisure sports.

## Figures and Tables

**Table 1:** Dimensions, items and statistics of constructs (*n* = 239).

Items		Mean	α	AVE	CR
**Participation**		11.435			
**Intrinsic Motivation**		4.077	0.792	0.501	0.798
	For the excitement I feel when I am really involved in running.	4.008			
	Because I feel a lot of satisfaction while mastering some running skills.	4.046			
	For the satisfaction I experience while I am perfecting my running abilities.	4.209			
	For the pleasure of acquiring new running knowledge and skills.	4.046			
**Constraints**		3.120	0.909	0.711	0.881
**Intrapersonal**		3.237	0.811	0.4707	0.815
	Physical injury	3.757			
	Security concerns	2.916			
	Negative state of mind	3.201			
	Lack of sports ability	2.921			
	Physical fatigue from running	3.385			
**Interpersonal**		2.928	0.811	0.606	0.8185
	Lack of running atmosphere	3.042			
	Lack of family support	2.749			
	Lack of suitable running friends	2.992			
**Structural**		3.165	0.780	0.486	0.787
	Lack of time	3.343			
	High money investment	2.778			
	Unfavorable weather	3.439			
	Lack of suitable places	3.100			
**Negotiation**		3.791	0.935	0.724	0.913
**Consciousness-raising**		3.915	0.800	0.566	0.797
	Learn knowledge	3.862			
	Strengthen willpower	3.975			
	Set goals	3.908			
**Self-management**		3.911	0.808	0.574	0.801
	Scientific training to improve running skills	3.929			
	Use professional sports equipment	3.908			
	Spend money	3.895			
**Helping relationships**		3.798	0.850	0.583	0.848
	Communicate with experienced runners	3.866			
	Get support of family	3.703			
	Join a running group	3.808			
	Get support and encouragement from running friends	3.816			
**Supportive environment**		3.667	0.806	0.596	0.813
	Change the running location	3.682			
	Change the running environment	3.770			
	Utilize the commute distance to run	3.548			

Note: α = Cronbach’s alpha; AVE = average variance extracted; CR = composite reliability.

**Table 2:** Correlation estimates of constructs.

	1	2	3	4
1. Intrinsic Motivation	**0.708**			
2. Constraints	−0.075	**0.843**		
3. Negotiation	0.569 **	−0.139 *	**0.851**	
4. Participation	0.215 **	−0.200 **	0.342 **	—

Note: The square root of each construct’s AVE is in the bold numbers; * *p* < 0.05; ** *p* < 0.01.

## Data Availability

The data presented in this study are available on request from the corresponding author. The data are not publicly available due to restrictions i.e., privacy or ethical.
